# Cyclo(l-Pro-l-Tyr) Isolated from the Human Skin Commensal *Corynebacterium tuberculostearicum* Inhibits Tyrosinase

**DOI:** 10.3390/ijms25137365

**Published:** 2024-07-04

**Authors:** Yuika Sekino, Ikuya Yamamoto, Masahiro Watanabe, Kouji Kuramochi, Yuuki Furuyama

**Affiliations:** Department of Applied Bioscience, Tokyo University of Science, 2641 Yamazaki, Noda 278-8519, Chiba, Japankuramoch@rs.tus.ac.jp (K.K.)

**Keywords:** tyrosinase inhibitor, skin microbiota, cyclic dipeptide, *Corynebacterium tuberculostearicum*

## Abstract

Melanin is produced by melanocytes to protect human skin from harmful ultraviolet radiation. During skin cell renewal, melanin and dead skin cells are disposed of. However, prolonged exposure to ultraviolet rays or aging can disturb this cycle, leading to skin hyperpigmentation due to melanin accumulation. Tyrosinase is a crucial enzyme involved in melanin biosynthesis. Although various compounds, including tyrosine inhibitors, that counteract melanin accumulation have been reported, some, such as hydroquinone, are toxic and can cause vitiligo. Meanwhile, the skin is the largest organ and the outermost layer of the immune system, containing a diverse range of bacteria that produce low-toxicity compounds. In the current study, we aim to identify metabolites produced by skin microbiota that inhibit tyrosinase. Specifically, mushroom tyrosinase served as the study model. Following commensal skin bacteria screening, *Corynebacterium tuberculostearicum* was found to inhibit tyrosinase activity. The active compound was cyclo(l-Pro-l-Tyr); commercially available cyclo(l-Pro-l-Tyr) also exhibited inhibitory activity. Docking simulations suggested that cyclo(l-Pro-l-Tyr) binds to the substrate-binding site of mushroom tyrosinase, obstructing the substrate pocket and preventing its activity. Hence, cyclo(l-Pro-l-Tyr) might have potential applications as a cosmetic agent and food additive.

## 1. Introduction

Exposure to ultraviolet (UV) light stimulates melanin synthesis in melanocytes, resulting in hyperpigmentation [1]. Melanin protects the skin from UV radiation. Meanwhile, excessive melanin accumulation can negatively affect the aesthetic appearance of the skin and reduce patients’ quality of life. Typically, melanin is removed from the skin cells through a 28-day turnover cycle. However, excessive exposure to UV light, aging, or other factors can disrupt this cycle, leading to melanin accumulation and skin blemishes [1]. Therefore, developing methods to control melanin production and prevent blemishing is necessary.

Tyrosinase is a crucial enzyme involved in melanin biosynthesis. l-tyrosine is oxidized to dihydroxyphenylalanine (DOPA) quinone via l-DOPA in a two-step reaction with tyrosinase [2,3]. DOPA quinone is converted into DOPA chrome via a spontaneous oxidation reaction, which is then polymerized to produce melanin [2,3]. In the cosmetic industry, tyrosinase inhibitors are used to suppress melanin production induced by overexposure to UV light or aging. Kojic acid, hydroquinone, and glutathione are well-known tyrosinase inhibitors commonly used as cosmetic ingredients owing to their anti-hyperpigmentation effects [3,4,5]. However, when applied topically, some of these compounds are toxic to human skin. For example, hydroquinone is not recommended due to its significant side effects, such as vitiligo-like symptoms, redness, and rash [4,6]. Therefore, tyrosinase inhibitors that effectively prevent excessive pigmentation without inducing adverse effects are needed.

Recently, there has been growing interest in identifying compounds derived from microorganisms as potential tyrosinase inhibitors [2,3]. For example, kojic acid, typically produced by the fungi *Aspergillus oryzae*, is a less harmful alternative to other synthetic tyrosinase inhibitors [3]. Indeed, soil and marine bacteria produce a diverse array of these inhibitors [2,3]. Moreover, probiotic bacteria such as *Lactobacillus* spp. and *Bifidobacterium* spp. exhibit tyrosinase inhibitory activity; however, the associated active compounds and underlying inhibitory mechanisms have not yet been elucidated. For example, while cyclo(-l-Pro-l-Tyr-l-Pro-l-Val-), produced by *Lactobacillus helveticus*, inhibits tyrosinase activity, the mechanism of action remains unknown [3]. Moreover, a diverse range of bacteria inhabit the human skin—the largest organ and the outermost layer of the immune system [7,8]. Given that dermal commensal bacteria are not targeted by the skin’s immune response, the compounds they produce are expected to have low toxicity to the skin [7,8]. However, to our knowledge, the production of tyrosinase inhibitors by skin bacteria has not yet been reported.

In this study, we aimed to identify tyrosinase inhibitors from human skin microbiota. To this end, more than 100 skin-derived bacterial stocks were obtained by swabbing and screened for tyrosinase inhibitory activity against *Agaricus bisporus* (mushroom) tyrosinase, which served as the model. Ultimately, *Corynebacterium tuberculostearicum* was found to inhibit tyrosinase via the active metabolite cyclo(l-Pro-l-Tyr).

## 2. Results

### 2.1. Corynebacterium tuberculostearicum Culture Broth Inhibited Mushroom Tyrosinase

**Figure 1:**
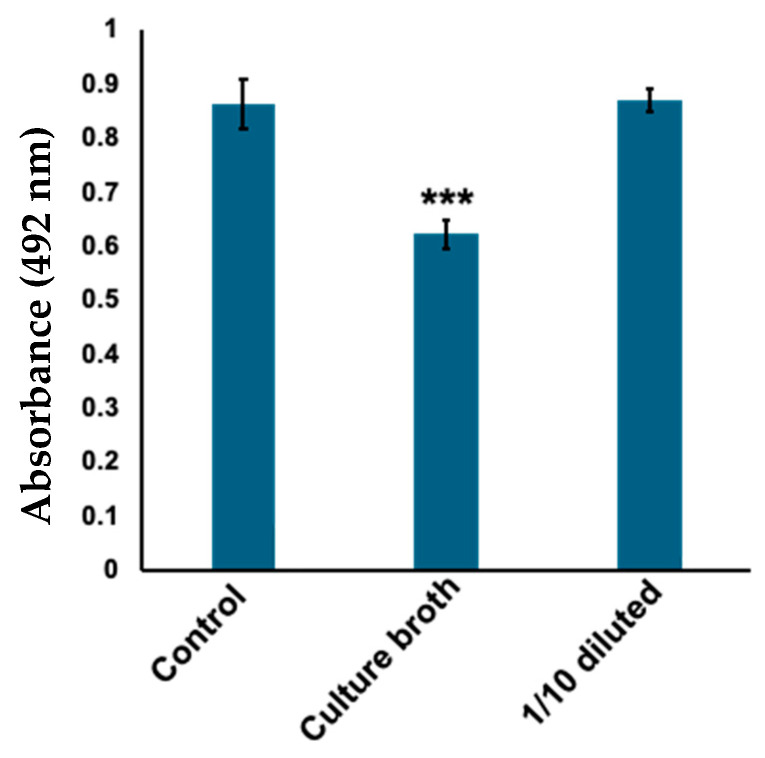
Culture broth with *Corynebacterium tuberculostearicum* inhibits tyrosinase activity. To evaluate the amount of DOPA chrome produced, absorbance was measured at 492 nm. Control: tyrosinase treated with water (negative control); culture broth: tyrosinase treated with filtered *C. tuberculostearicum* culture broth; 1/10 diluted: tyrosinase treated with ten times diluted *C. tuberculostearicum* broth. Bars: standard error (SE), *** *p* < 0.001.

Skin microbiota stocks were randomly selected and cultured overnight in Gifu Anaerobic Medium (GAM) liquid medium under aerobic conditions. The culture broths were filtered through a 0.22 μm filter and screened based on tyrosinase inhibitory activity. The tyrosinase activity was evaluated in terms of DOPA chrome production from l-DOPA by measuring absorbance at 492 nm. We found that a sample of sterile culture supernatant significantly inhibited the formation of DOPA chrome by tyrosinase (Figure 1). Phylogenetic analysis using 16S rRNA sequencing revealed that the strain exhibiting tyrosinase inhibitory activity was *C. tuberculostearicum*.

### 2.2. Ethyl Acetate Extract Exhibited Tyrosinase Inhibitory Activity

**Figure 2:**
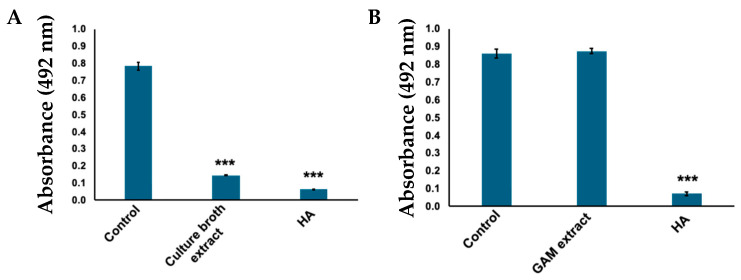
Tyrosinase inhibitory activity of ethyl acetate extracts. DOPA chrome produced was evaluated by measuring absorbance at 492 nm. (**A**) Absorbance when tyrosinase was treated with *C. tuberculostearicum* broth ethyl acetate extract; bars: standard deviation (SD), *** *p* < 0.001. (**B**) Gifu Anaerobic Medium (GAM) plate extract showing no inhibitory activity to tyrosinase. Control: tyrosinase treated with water (negative control); HA: tyrosinase treated with 2 mM homogentisic acid (positive control); bars: standard deviation (SD), *** *p* < 0.001.

Considering that *C. tuberculostearicum* metabolites showed inhibitory activity against tyrosinase (Figure 1), the culture medium was extracted with ethyl acetate to obtain the active compound. The ethyl acetate extract also exhibited tyrosinase inhibitory activity (Figure 2A). Furthermore, no activity was observed when only the GAM medium was extracted (Figure 2B).

### 2.3. Purification of the Active Compound from C. tuberculostearicum Culture Broth

**Figure 3:**
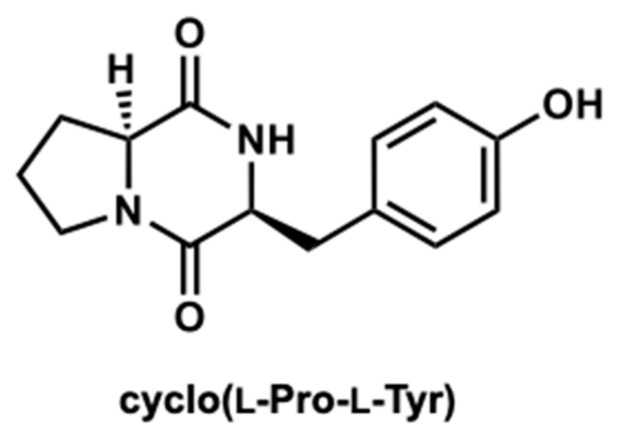
Structure of cyclo(l-Pro-l-Tyr).

**Figure 4:**
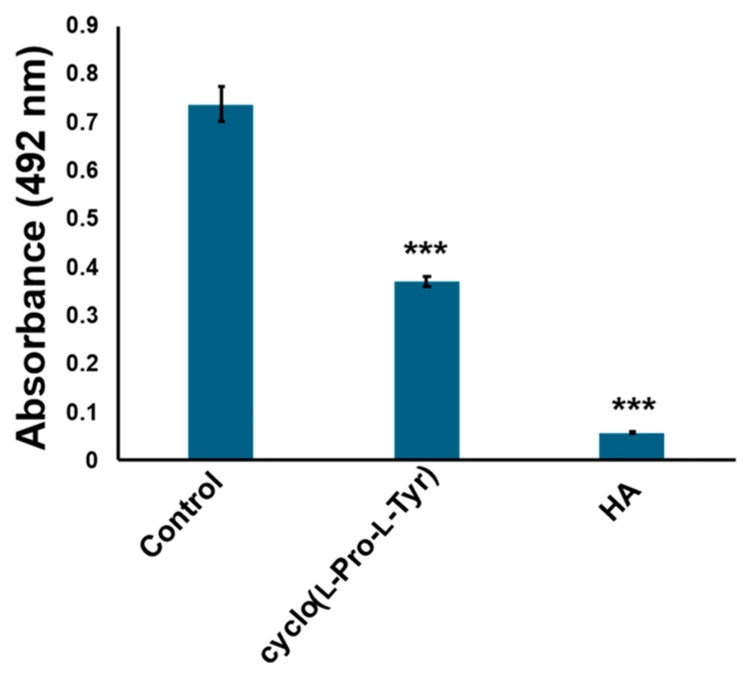
Cyclo(l-Pro-l-Tyr) isolated from culture broth inhibits tyrosinase activity. Tyrosinase activity was evaluated by measuring absorbance at 492 nm. Cyclo(l-Pro-l-Tyr): tyrosinase treated with 1 mg/mL purified cyclo(l-Pro-l-Tyr); control: tyrosinase treated with phosphate-buffered saline (PBS) (negative control); HA: tyrosinase treated with 2 mM homogentisic acid (positive control); bars: standard deviation (SD), *** *p* < 0.001.

**Figure 5:**
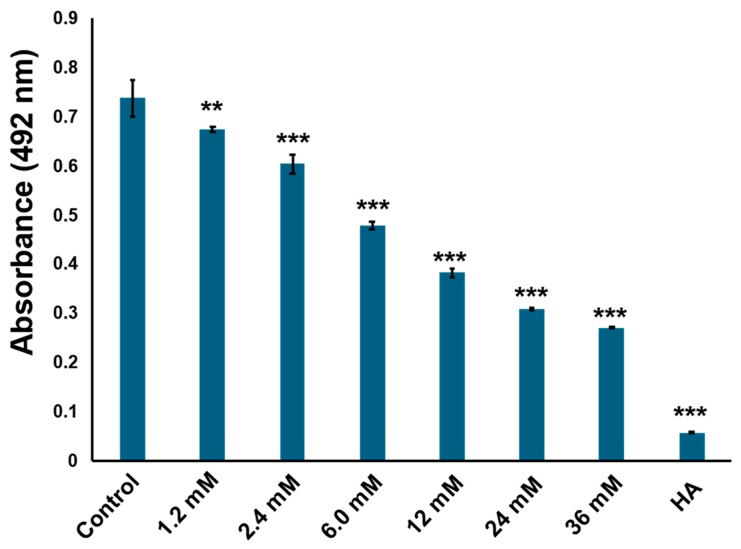
Commercially available cyclo(l-Pro-l-Tyr) inhibits tyrosinase activity in a concentration-dependent manner. Tyrosinase activity was evaluated by measuring absorbance at 492 nm. Control: tyrosinase treated with phosphate-buffered saline (PBS) (negative control); HA: tyrosinase treated with 2 mM homogentisic acid (positive control); bars: standard deviation (SD), ** *p* < 0.01, *** *p* < 0.001.

The compounds were further purified using preparative thin layer chromatography (Figure S1). The structure was determined as cyclo(l-Pro-l-Tyr) by its ^1^H and ^13^C nuclear magnetic resonance (NMR) spectroscopic and specific rotation data (Figure 3 and Figure S2). Data of commercially available cyclo(l-Pro-l-Tyr) corresponded with the obtained data (Figures S3 and S4). Purified cyclo(l-Pro-l-Tyr) exhibited inhibitory activity against mushroom tyrosinase (Figure 4). To verify the activity of cyclo(l-Pro-l-Tyr), the tyrosinase inhibitory activity of commercially available cyclo(l-Pro-l-Tyr) was tested. Purchased cyclo(l-Pro-l-Tyr) exhibited tyrosinase inhibitory activity (Figure 5) with a *K*i value of 9.86 mM as substrate for l-DOPA (Figure S5). The structure of the compound in another fraction was found to be adenine (Figures S1, S6 and S7). Purchased adenine did not show tyrosinase inhibitory activity (Figure S8).

### 2.4. Cyclo(l-Pro-l-Val) and Cyclo(l-Pro-l-Leu) Dose Not Inhibit Tyrosinase Activity

**Figure 6:**
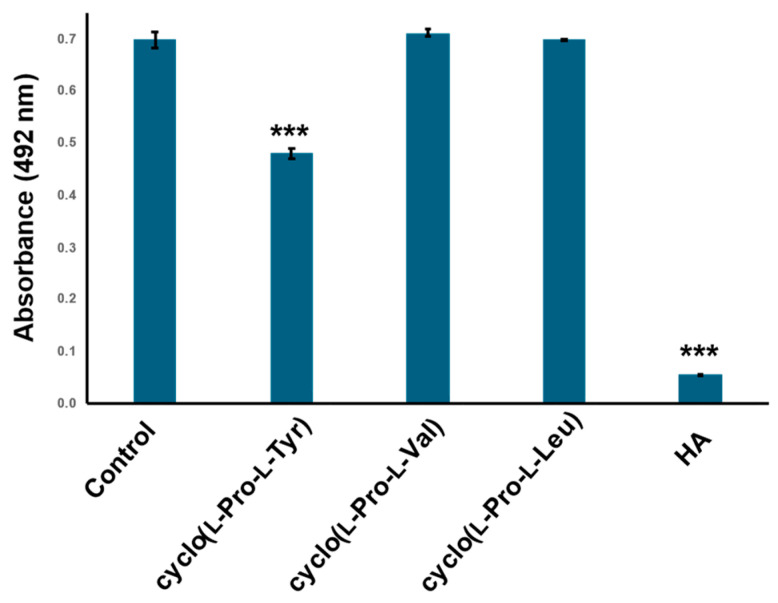
Cyclo (l-Pro-l-Val) and cyclo(l-Pro-L-Leu) did not show tyrosinase inhibitory activity. Tyrosinase activity was evaluated by measuring absorbance at 492 nm. Commercially available cyclo(l-Pro-l-Tyr), cyclo (l-Pro-l-Val) and cyclo(l-Pro-L-Leu) were treated with 12 mM tyrosinase. Control: tyrosinase treated with phosphate-buffered saline (PBS) (negative control); HA: tyrosinase treated with 2 mM homogentisic acid (positive control); bars: standard deviation (SD), *** *p* < 0.001.

To establish whether the tyrosine residue of cyclo(l-Pro-l-Tyr) is important for inhibitory activity, tyrosinase was treated with two analogs, cyclo(l-Pro-l-Val) and cyclo(l-Pro-l-Leu), both of which did not inhibit tyrosinase activity (Figure 6).

### 2.5. Cyclo (l-Pro-l-Tyr) Bound to Tyrosinase Substrate-Binding Site and Competitively Inhibited Its Enzymatic Activity

**Figure 7:**
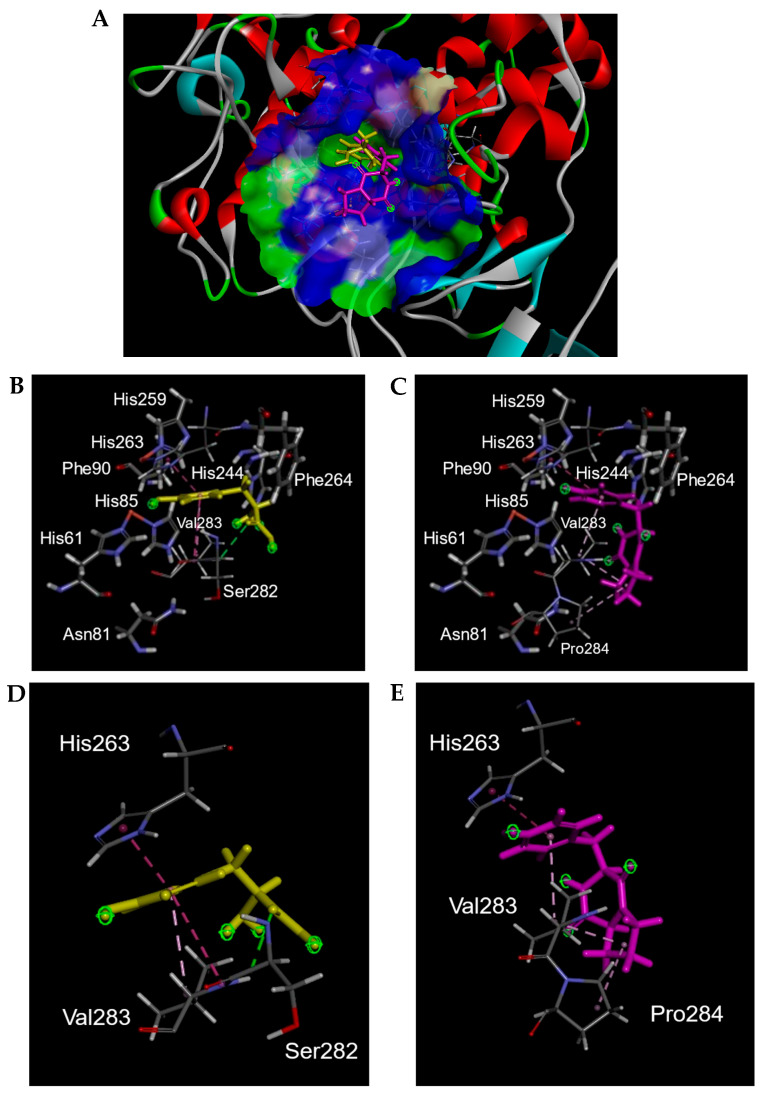
Three-dimensional (3D) plots of molecular docking diagrams of l-tyrosine and cyclo(l-Pro-l-Tyr) complexes with tyrosinase. (**A**) These two compounds are indicated with tyrosinase. Yellow: Tyr; light purple: l-Pro-l-Tyr; blue and green areas indicate solvent-accessible surfaces of tyrosinase. Tyrosinase is indicated with red, gray, cyan, and green ribbons. Both l-tyrosine and cyclo(l-Pro-l-Tyr) were positioned in the substrate-binding pocket. Interaction of (**B**,**D**) l-tyrosine and (**C**,**E**) cyclo(l-Pro-l-Tyr) with tyrosinase in the substrate-binding pocket. (**B**,**C**) Amino acids comprising the substrate binding pocket are shown. (**D**,**E**) Only amino acids interacting with l-tyrosine or cyclo(l-Pro-l-Tyr) are indicated. Yellow: Tyr; light purple: l-Pro-l-Tyr.

To predict the mode of action of cyclo(l-Pro-l-Tyr), docking simulations of tyrosinase and cyclo(l-Pro-l-Tyr) were performed using Discovery Studio 2022. When l-tyrosine was used as the substrate, we obtained 10 conformation candidates (Figures S9–S18). Based on previous reports regarding the binding modes of l-tyrosine or inhibitors with tyrosinase [9,10,11,12,13], the conformation of l-tyrosine binding to the tyrosinase active site via a benzene ring (Pose No. 10) was used in this study (Figure 7A,B,D and Figure S18). In Figure 7A, the molecule docking results of l-tyrosine and cyclo(l-Pro-l-Tyr) with tyrosinase are shown, suggesting that cyclo(l-Pro-l-Tyr) bound to the tyrosinase active site and occupied the substrate pocket. In the substrate-binding pocket, l-tyrosine interacted with His263, Ser282, and Val283 (Figure 7B,D). Cyclo(l-Pro-l-Tyr) also bound to the same pocket and interacted with His263 and Val283 (Figure 7C,E). Additionally, the proline residue of cyclo(l-Pro-l-Tyr) interacted with Pro284 at the entrance of the substrate pocket instead of Ser282 (Figure 7E). These results indicated that Cyclo(l-Pro-l-Tyr) inhibits tyrosinase activity in a competitive manner. In our docking study, the CDOCKER energy [14] between the mushroom tyrosinase and l-tyrosine, cyclo(l-Pro-l-Tyr) was found to be −24.0 kcal/mol and −11.4 kcal/mol, respectively.

## 3. Discussion

*Corynebacterium tuberculostearicum* widely colonizes the human skin and is preferentially abundant in moist areas [15,16]. To our knowledge, this is the first report of *C. tuberculostearicum* exhibiting tyrosinase inhibitory activity and producing cyclo(l-Pro-l-Tyr). Although the activity identified in this study was not very strong, our results suggest that the amount of cyclo(l-Pro-l-Tyr) contained in the *C. tuberculostearicum* culture medium was high. As this bacterium resides on the skin, it is expected to exert low toxicity in humans. In fact, cyclo(l-Pro-l-Tyr) is nontoxic to normal human cells [17,18]. Therefore, this bacterium has potential utility as a skin probiotic to combat hyperpigmentation.

Cyclo(l-Pro-l-Tyr), also known as maculosin-1, is a host-specific toxin produced by *Alternaria alternata*, a filamentous phytopathogenic fungus [19]. The production of this compound by other microorganisms, such as *Lysobacter* sp., *Streptomyces* sp., *Bacillus* sp., and *Pseudomonas* sp., has also been reported [17,20,21,22]. In addition to phytotoxin activity, antifungal, antibacterial, antioxidant, and anticancer activities have also been reported [17,20,21,22].

The ethyl acetate extract showed strong inhibitory activity comparable with that of homogentisic acid. However, the activity of purified cyclo(l-Pro-l-Tyr) slightly reduced, possibly owing to material loss during the purification process or in the culture broth, as there are other active compounds that exert a stronger inhibitory activity. As shown in Figure 5, the initial ethyl acetate extract contained more than 36 mM cyclo(l-Pro-l-Tyr). The synthesis of cyclic peptides, including cyclo(l-Pro-l-Tyr), is a complex process [23]. Cyclic peptides are important synthetic targets in the pharmaceutical industry because of their various activities [13]. The discovery that a significant amount of cyclo(l-Pro-l-Tyr) could be extracted from *C. tuberculostearicum* is expected to support the industrial production of cyclo(l-Pro-l-Tyr).

His61, His85, His94, His259, His263, and His296 of mushroom tyrosinase, PPO3, are important amino acids that form substrate pockets; His263 is particularly involved in tyrosine binding [24,25,26,27]. In silico molecular docking simulations indicated that cyclo(l-Pro-l-Tyr) enters the substrate pocket and binds to His263 and other amino acids, such as Val283, in the substrate pocket. The CDOCKER energy of this compound was −11.4 kcal/mol, and it was assumed that this compound competitively inhibited tyrosinase activity. The fact that cyclo(l-Pro-l-Val) and cyclo(l-Pro-L-Leu) did not inhibit tyrosinase supports this docking model.

Tyrosinase inhibitors can be used to prevent age spots [3] and treat malignant melanoma [28]. Furthermore, not only for skin issues, but tyrosinase inhibitors can also be used to treat neurological diseases such as Parkinson’s disease [3]. In the future, further evaluation of human-derived tyrosinases and a more detailed analysis of their mechanisms of action are required. Human and mushroom tyrosinases have approximately 20% homology, and compounds that are effective against mushroom tyrosinases may not be effective against human tyrosinase [29,30]. Microorganism-induced melanin synthesis can lead to discoloration in plants, including food crops, and mushroom tyrosinase inhibitors have been shown to prevent discoloration [3,30]. Thus, cyclo(l-Pro-l-Tyr) also has potential applications as a pesticide and food additive.

## 4. Materials and Methods

### 4.1. Isolation and Culture of Skin Bacteria

Skin bacteria were sampled from the faces of the two males in their 20’s and one female in her 20’s using the swabbing method and cultured on a GAM plate (GAM broth; Nissui Co., Tokyo, Japan; 1.5% agar) at 37 °C under aerobic or anaerobic conditions for isolation. This resulted in the isolation of 180 bacterial samples. The isolated bacteria were cultured overnight in GAM broth at 37 °C and 138 rpm under aerobic conditions. To use the tyrosinase inhibitory assay, the culture broth of skin bacteria was sterilized with a 0.22 µm filter (Millipore, Bedford, MA, USA).

### 4.2. Identification of the Active Strain

The active strain was identified using 16S rRNA sequencing. The 16S rRNA amplification was performed by PCR using KOD Fx Neo (TOYOBO, Tokyo, Japan) and two universal primers, 27F and 1492R. They were then sequenced using the universal primers 518F and 800R. The 16S rRNA sequences were determined using the Basic Local Alignment Search Tool. A comparison of gene data indicated that the active strain was *C. tuberculostearicum.*

### 4.3. Tyrosinase Inhibitory Assay

Tyrosinase inhibition was evaluated by monitoring the formation of DOPA chrome from l-DOPA. The amount of converted DOPA chrome was estimated by measuring absorbance at 492 nm. Mushroom (*Agaricus bisporus*) tyrosinase (Worthington Biochemical Corporation, Lakewood, NJ, USA, Cat. No. LS003792, Lot: 32M22876) and l-DOPA (Nacalai Tesque Inc., Kyoto, Japan) were dissolved in PBS (1 mM sodium phosphate in distilled water, pH = 6.8), respectively, to obtain the l-DOPA solution (300 µg/mL) and the tyrosinase solution (100 µg/mL). The filtered skin bacterial culture broth was serially diluted with GAM medium (1/10, 1/100, 1/1000). For inhibition assays, diluted broth (80 µL) at each concentration was mixed with tyrosinase solution (80 µL) in a 96-well plate and heated at 37 °C for 10 min. The l-DOPA solution (80 µL) was then added to each well, mixed, and incubated at 37 °C for 5 min. The absorbance was measured at 492 nm. A reaction solution with 80 µL PBS instead of filtered broth was used as the negative control, and homogentisic acid (2 mM) was used as the positive control [31]. For the ethyl acetate extract of the test, 80 µL extract (3.4 × 10^3^ µg/mL) was added to the reaction solution. For the cyclo(l-Pro-l-Tyr) assay, isolated cyclo(l-Pro-l-Tyr) (1 mg/mL) or purchased cyclo(l-Pro-l-Tyr) (1.2, 2.4, 6, 12, 24, and 36 mM) were prepared in PBS and added to the reaction solution. Cyclo(l-Pro-l-Val) was purchased from Bachem AG (Bubendorf, Switzerland) and Cyclo(l-Pro-l-Leu) was purchased from Tokyo Kasei Kogyo (TCI) (Tokyo, Japan), and treated in the same manner as above.

### 4.4. Kinetic Analysis of Tyrosinase Inhibition

To determine the kinetic mechanism, Dixon plots were used. A plot of 1/enzyme velocity (1/v) was plotted against the inhibitor concentration (I) to determine the Ki value for the enzyme–inhibitor complex. Dixon plots of the inhibition were obtained in the presence of l-DOPA substrate at 300 and 600 µg/mL, and 0, 1.2, 2.4, 6.0, 12, 24, and 36 mM for cyclo(l-Pro-l-Tyr).

### 4.5. Extraction, Purification, and Structural Identification of Cyclo(l-Pro-l-Tyr)

The *C. tuberculostearicum* culture broth was extracted using ethyl acetate and concentrated to obtain a crude extract for the tyrosinase inhibitory assay. The extracted sample was purified using preparative thin layer chromatography (PLC Silica gel 60 F_254_, 0.5 mm) with CHCl_3_/MeOH (10:1), and the compounds were detected using UV (254 nm). NMR spectra and optical spectra indicated that the active compound was cyclo(l-Pro-l-Tyr).

Specific rotations were recorded on a JASCO polarimeter (P-2200) as [α]_D_ values (concentration in g/100 mL). NMR spectra were recorded on a Bruker Avance 400 spectrometer at 400 MHz for ^1^H NMR and at 100 MHz for ^13^C NMR using methanol-*d*_4_ (MeOD) as the solvent.

For cyclo(l-Pro-l-Tyr) purified from culture broth; [α]_D_^27^ = −37.9 (*c* 0.12, EtOH); ^1^H NMR (400 MHz, MeOD), δ_H_: 7.03 (2H, d, *J* = 8.4 Hz), 6.69 (2H, d, *J* = 8.4 Hz), 4.36 (1H, m), 4.04 (1H, ddd, *J* = 10.8, 6.4, 1.6 Hz), 3.48–3.58 (1H, m), 3.05 (2H, ABqd, *J* = 12.0, 4.8 Hz), 2.05–2.11 (2H, m), 1.76–1.83 (2H, m); and ^13^C NMR (100 MHz, MeOD), δ_C_: 170.8, 167.0, 157.7, 132.1 (2C), 127.6, 116.2 (2C), 60.1, 57.9, 45.9, 37.7, 29.4, 22.7.

### 4.6. Molecular Docking Simulation

In silico molecular docking models were constructed using the Discovery Studio v 24.1.100.2404 software (Accelrys Co., Ltd., San Diego, CA, USA). The receptor protein PPO3 (PDB ID: 2Y9X) was obtained from the Protein Data Bank (PDB) (http://www.rcsb.org, accessed on 21 March 2023), providing the three-dimensional structure file of the protein. The water molecules on the tyrosinase were removed, followed by the addition of polar hydrogen atoms. l-tyrosine and cyclo(l-Pro-l-Tyr) were constructed using Chem Draw 17.0 (Cambridge, UK) and used as ligands, and the structure was optimized using Discovery Studio. The CDOCKER docking programs implemented in Discovery Studio were used. “Pose Cluster Radius” parameter and “Random Confirmation” parameter were set to 0.5 and 30, respectively; other parameters were set to default values.

### 4.7. Statistical Analysis

All experimental results are expressed as the mean ± standard deviation of three replicates. Differences were analyzed using Dunnett’s test to determine differences between experimental samples (triplicate) using Microsoft Office Excel (version 2406)and R software (version 4.0.2, R Foundation for Statistical Computing, Austria).

## Data Availability

The original contributions presented in the study are included in the article and Supplementary Materials. Further inquiries can be directed to the corresponding author.

## Supplementary Materials

The following supporting information can be downloaded at: https://www.mdpi.com/article/10.3390/ijms25137365/s1.
