# Supplementary material for: Cyclo(l-Pro-l-Tyr) Isolated from the Human Skin Commensal Corynebacterium tuberculostearicum Inhibits Tyrosinase
Source: Int J Mol Sci. 2024 Jul 4;25(13):7365. doi: 10.3390/ijms25137365 (PMC11242031; doi:10.3390/ijms25137365)
Supplement: Supplementary file 1 [file ijms-25-07365-s001.zip › ijms-3035251-supplementary.pdf]

*Supplementary Materials*

# Cyclo(L-Pro-L-Tyr) Isolated from the Human Skin Commensal *Corynebacterium tuberculostearicum* Inhibits Tyrosinase

**Yuika Sekino, Ikuya Yamamoto, Masahiro Watanabe, Kouji Kuramochi and Yuuki Furuyama***

Department of Applied Bioscience, Tokyo University of Science, 2641 Yamazaki, Noda, Chiba 278-8519, Japan

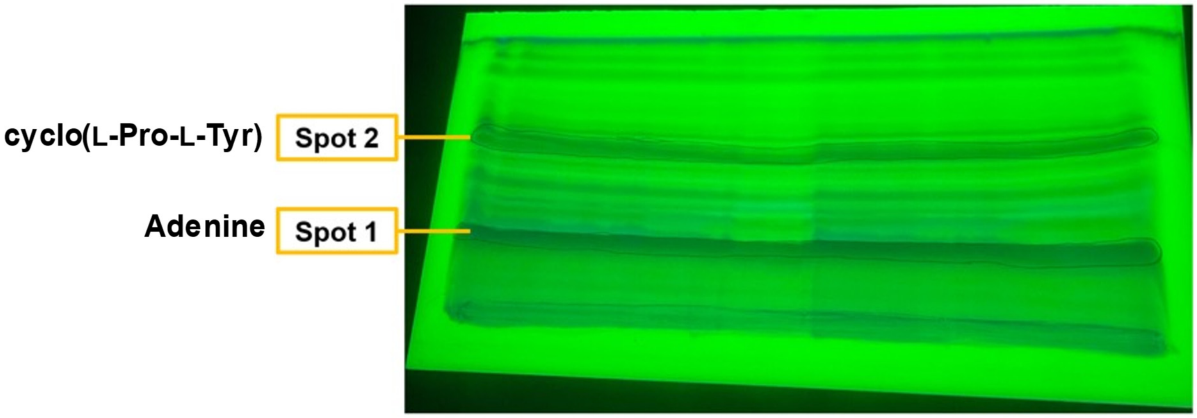


**Figure S1.** Image of preparative thin layer chromatography.

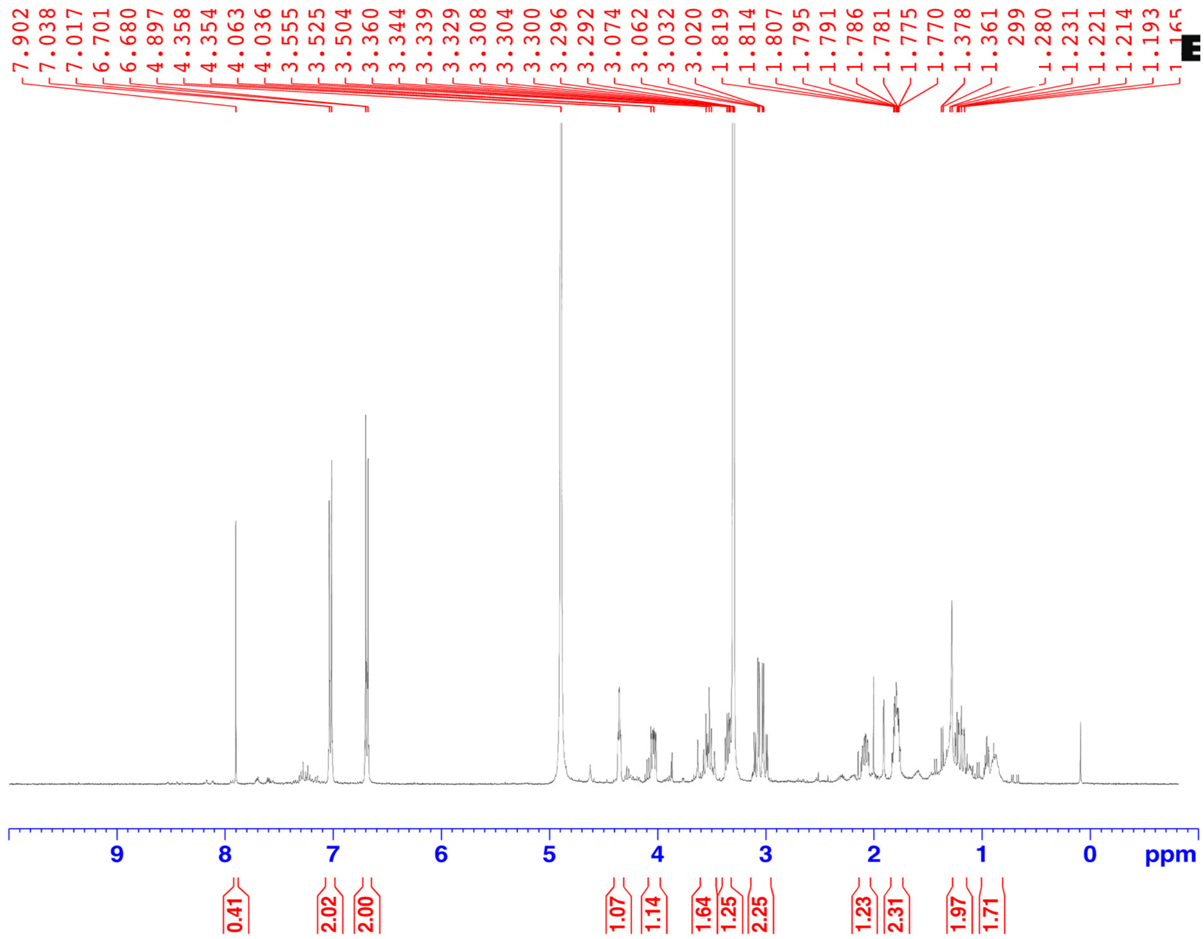


**Figure S2.** $^{1}$H NMR (400 MHz, MeOD) spectra of isolated cyclo(L-Pro-L-Tyr).

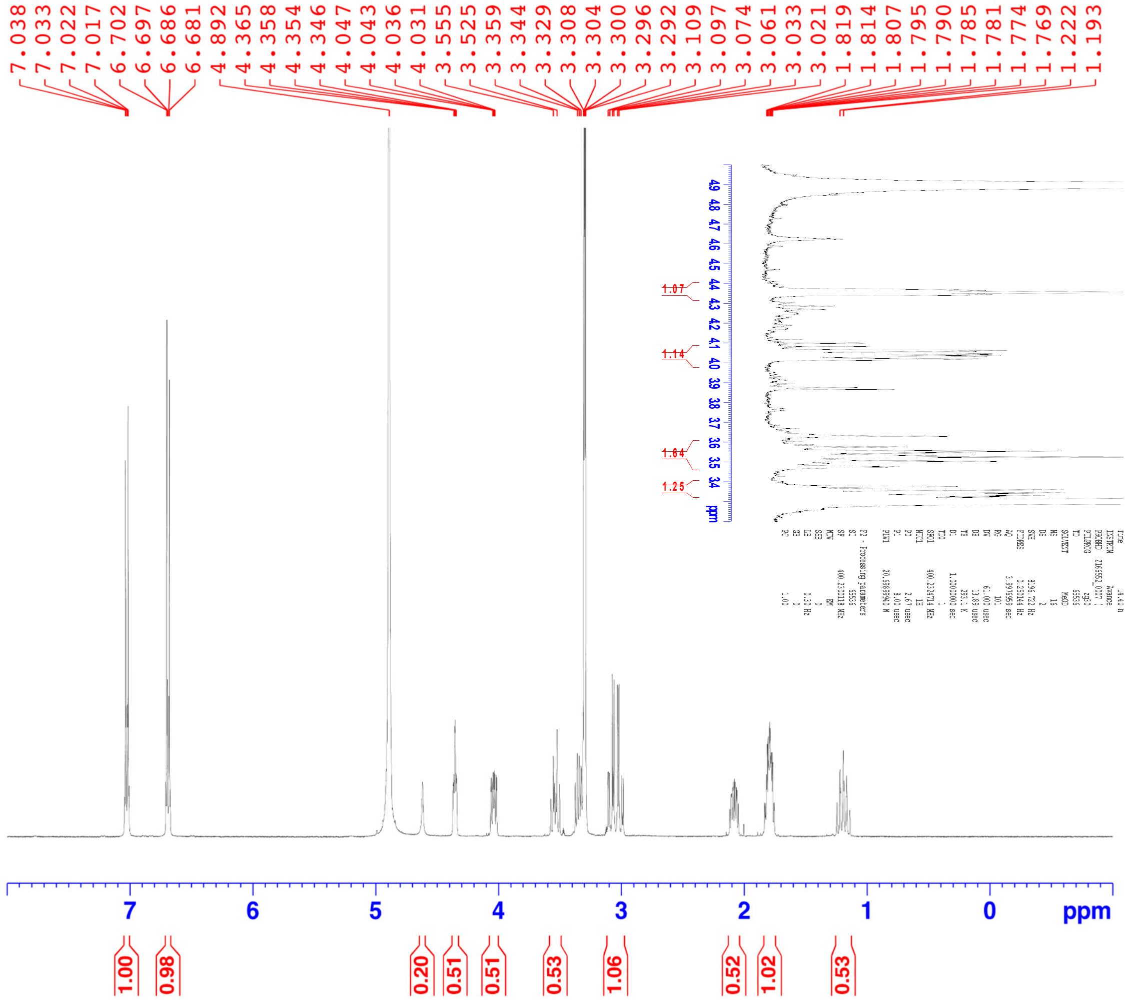

**Figure S3.** $^{1}H$ NMR (400 MHz, MeOD) spectra of commercially available cyclo(L-Pro-L-Tyr). Insert is extended figure (3.2 ppm to 5.0 ppm).

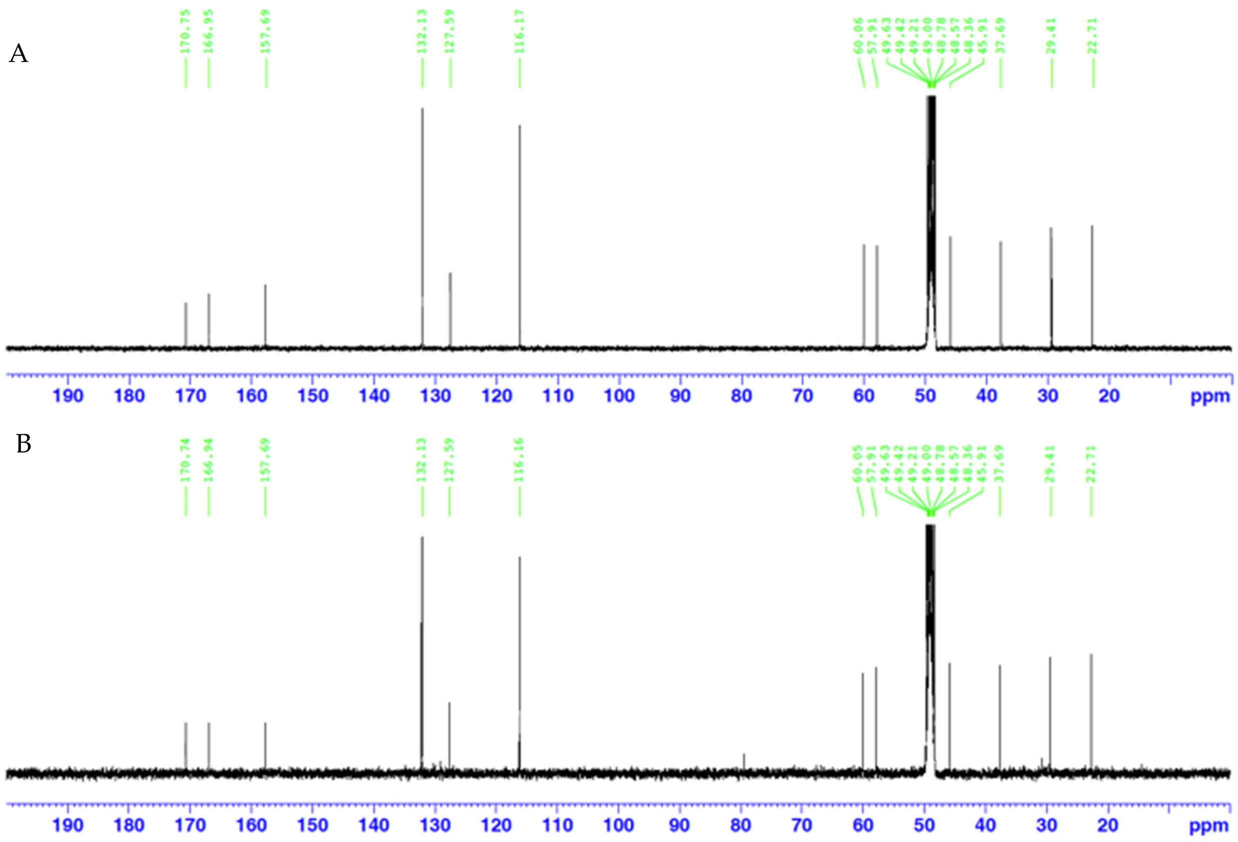


**Figure S4.** $^{13}C$ NMR (100 MHz, MeOD) spectra of isolated cyclo(L-Pro-L-Tyr) and commercially available cyclo(L-Pro-L-Tyr). (A) isolated cyclo(L-Pro-L-Tyr); (B) commercially available cyclo(L-Pro-L-Tyr).

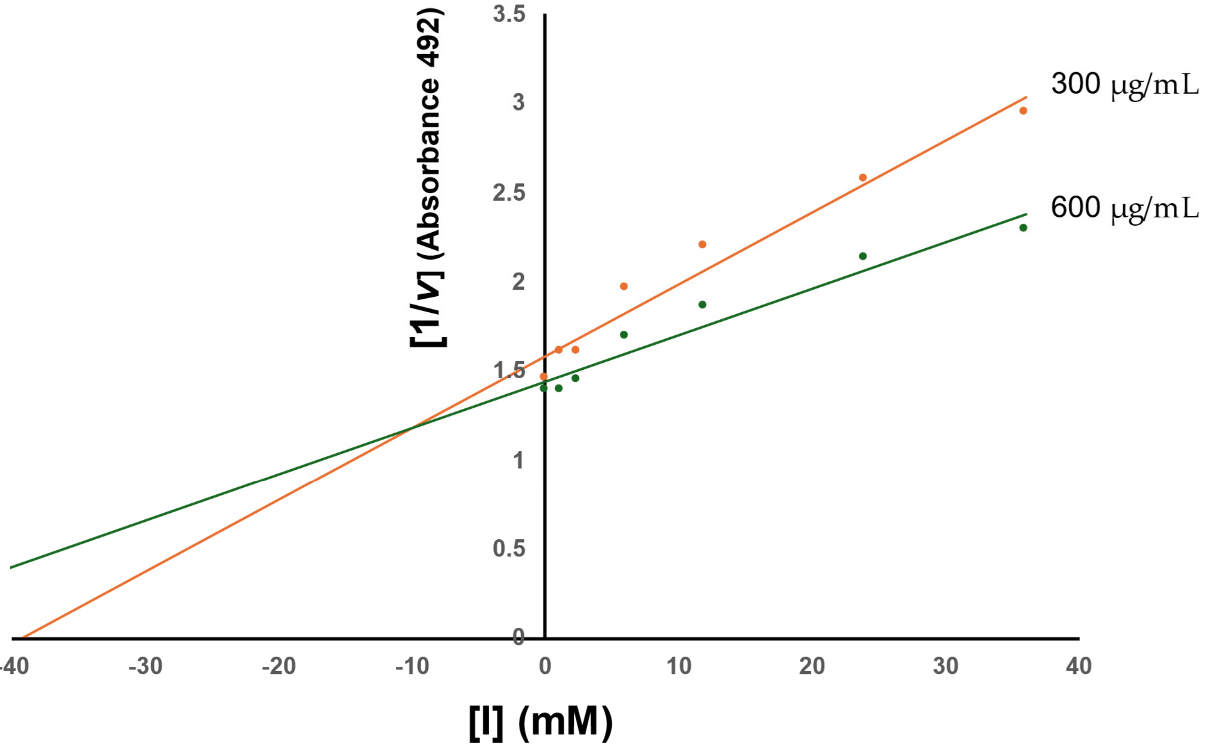


**Figure S5.** Dixon plot for tyrosinase enzyme inhibition by cyclo(L-Pro-L-Tyr).

A

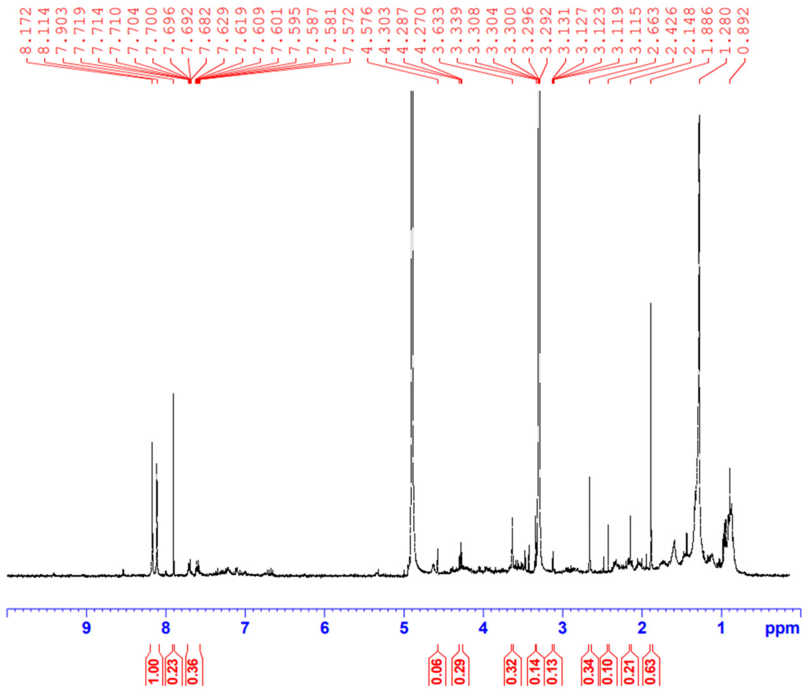


B

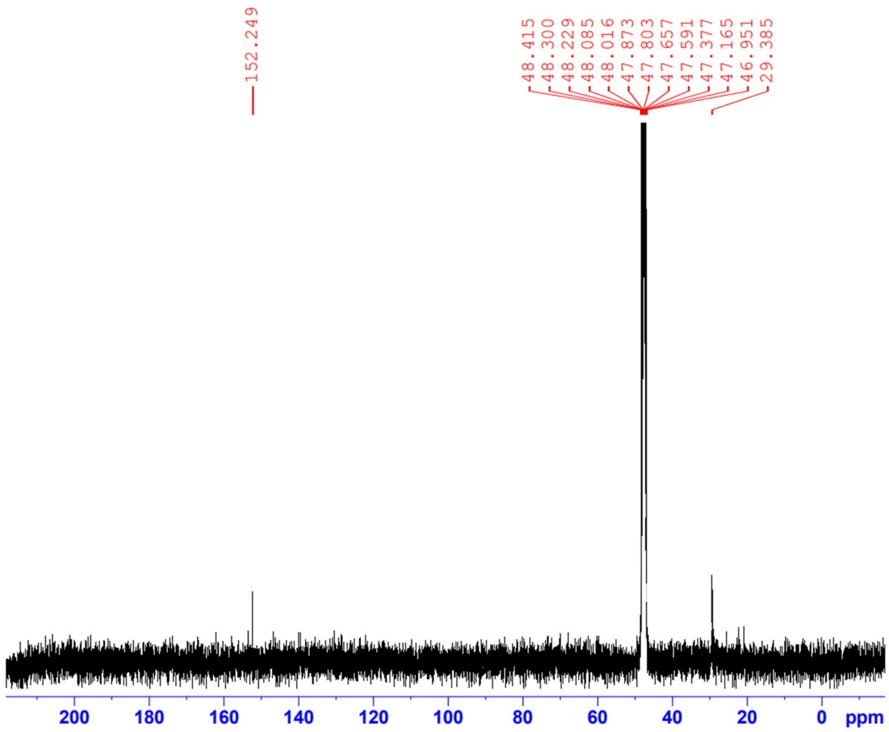


**Figure S6.** NMR spectrum of Spot 1 in Figure S14. (A) $^{1}H$ NMR (400 MHz, MeOD), (B) $^{13}C$ NMR (100 MHz, MeOD).

A

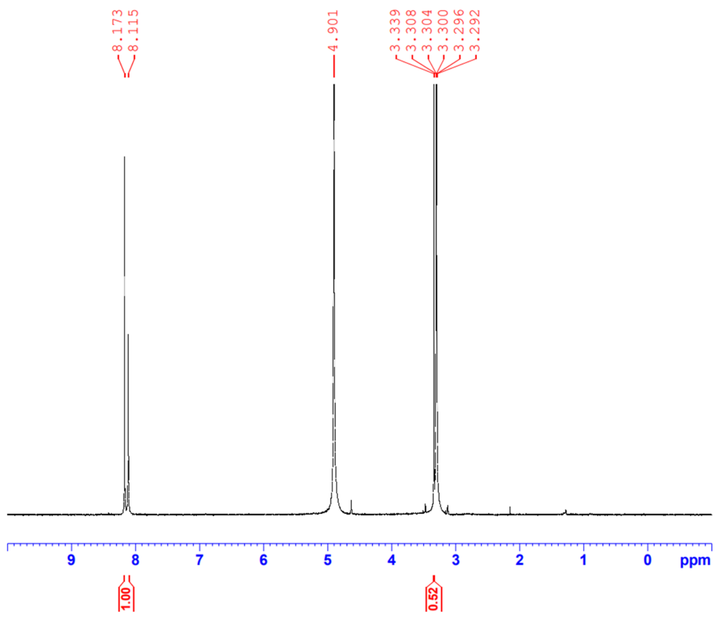


B

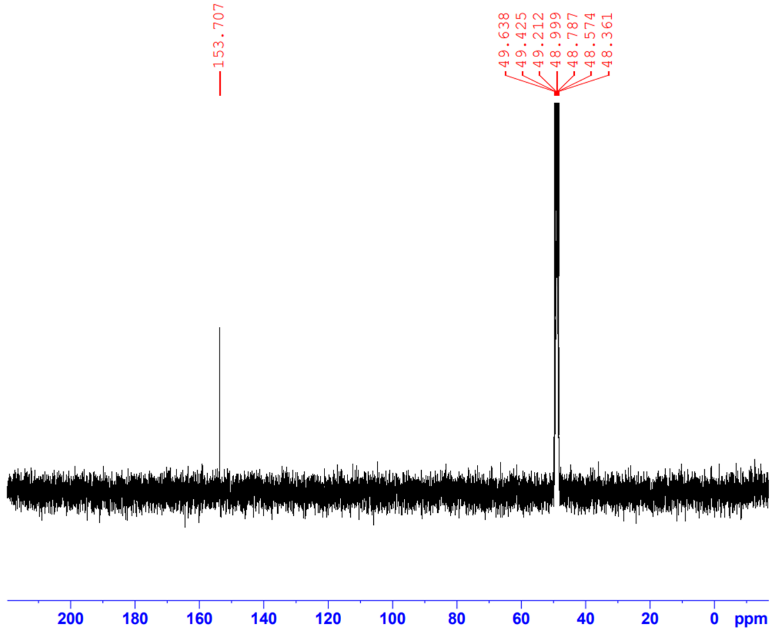


**Figure S7**. NMR spectrum of commercially available adenine. (A) $^{1}H$ NMR (400 MHz, MeOD), (B) $^{13}C$ NMR (100 MHz, MeOD).

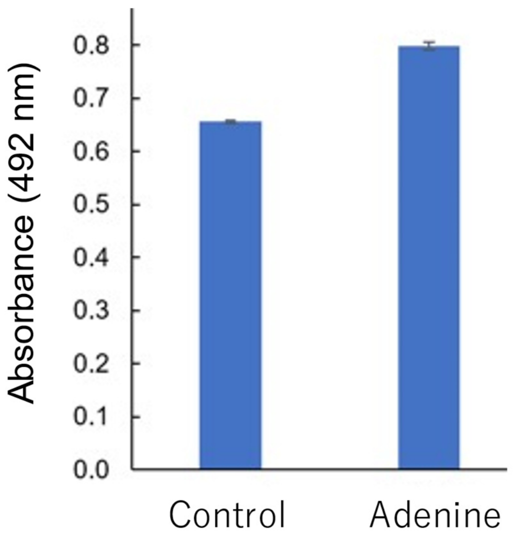


**Figure S8.** Commercially available adenine does not exhibit tyrosinase inhibitory activity. Tyrosinase activity was evaluated based on absorbance at 492 nm. Commercially available adenine was treated with 1 mg/mL tyrosinase; control: tyrosinase treated with water (negative control); Bars: standard deviation (SD). Adenine was purchased from FUJIFILM Wako Pure Chemicals (Tokyo, Japan).

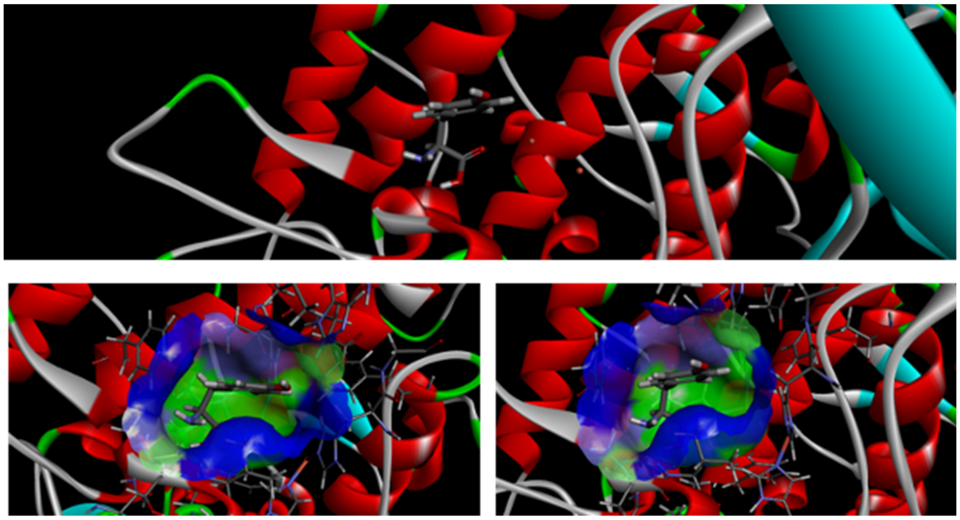

**Figure S9**. Pose No. 1 of tyrosine and tyrosinase docking. Red points: Cu; blue and green areas: solvent-accessible surfaces; red, gray, cyan, and green ribbons: tyrosinase.

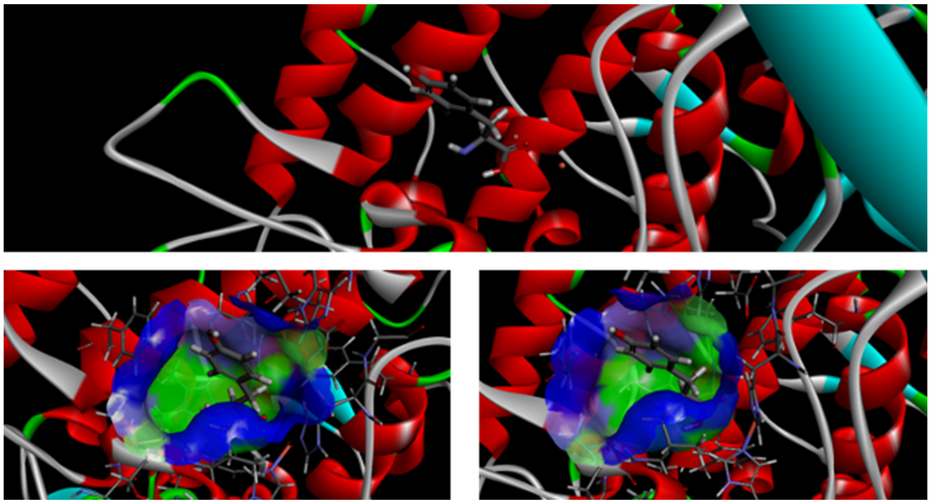

**Figure S10**. Pose No. 2 of tyrosine and tyrosinase docking. Red points: Cu; blue and green areas: solvent-accessible surfaces; red, gray, cyan, and green ribbons: tyrosinase.

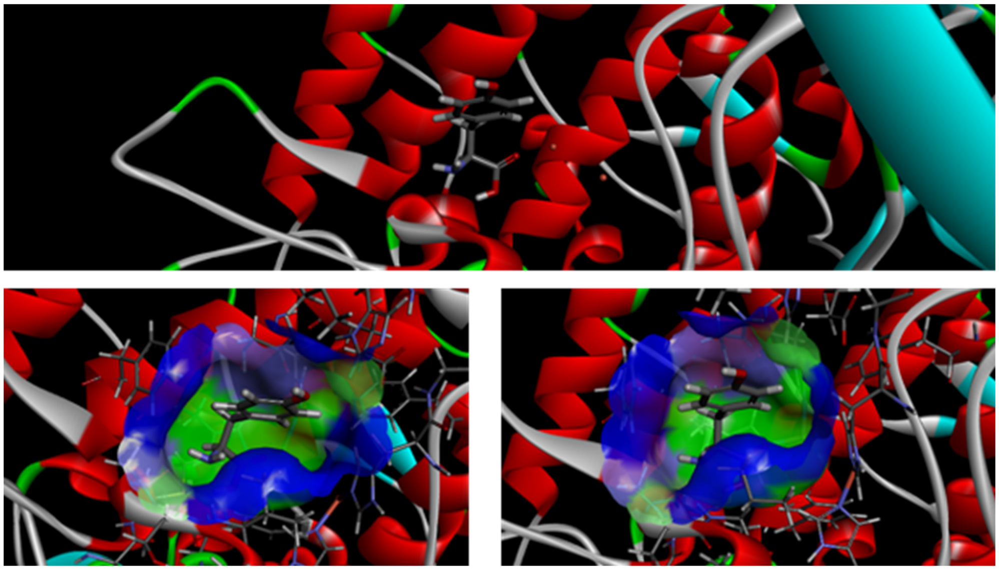

**Figure S11**. Pose No. 3 of tyrosine and tyrosinase docking. Red points: Cu; blue and green areas: solvent-accessible surfaces; red, gray, cyan, and green ribbons: tyrosinase.

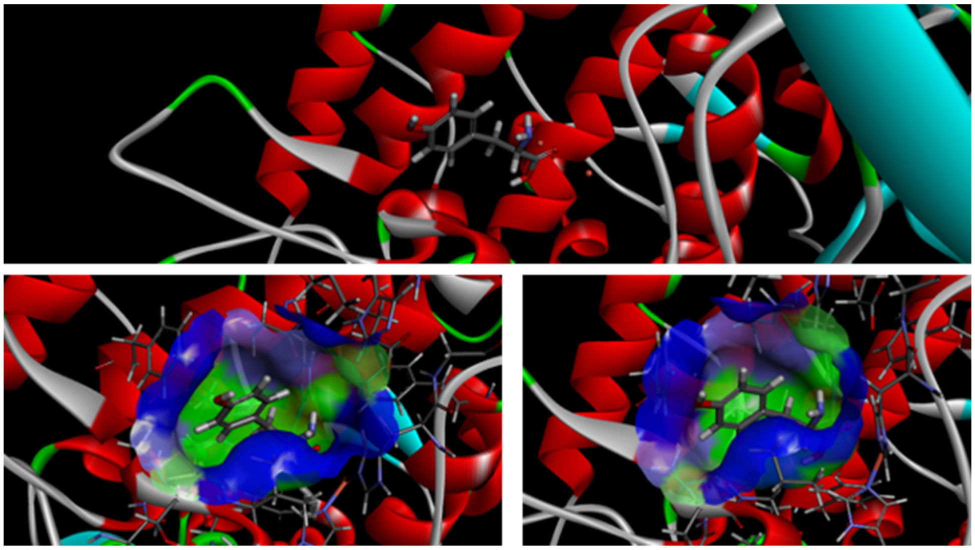

**Figure 12**. Pose No. 4 of tyrosine and tyrosinase docking. Red points: Cu; blue and green areas: solvent-accessible surfaces; red, gray, cyan, and green ribbons: tyrosinase.

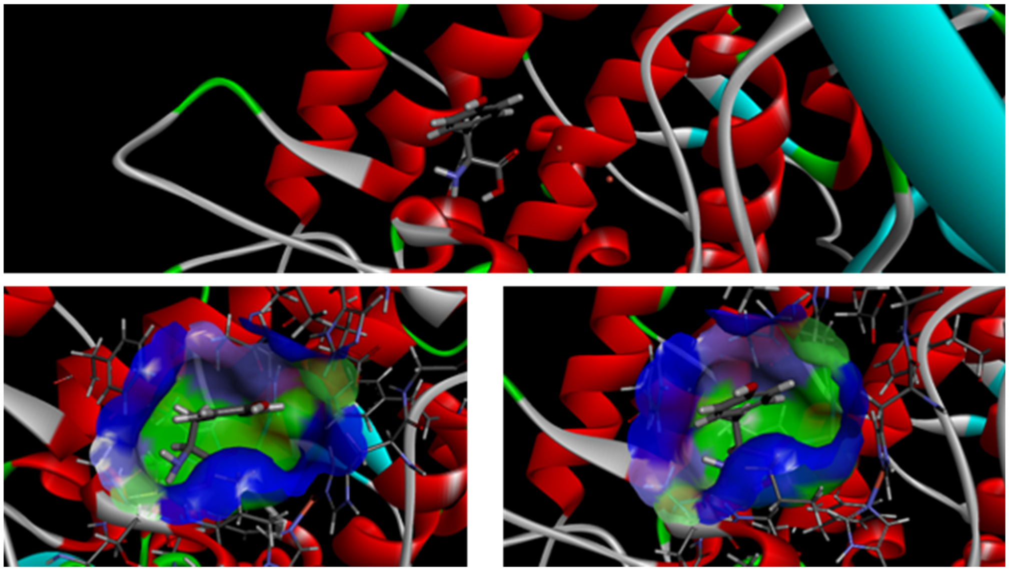

**Figure S13**. Pose No. 5 of tyrosine and tyrosinase docking. Red points: Cu; blue and green areas: solvent-accessible surfaces; red, gray, cyan, and green ribbons: tyrosinase.

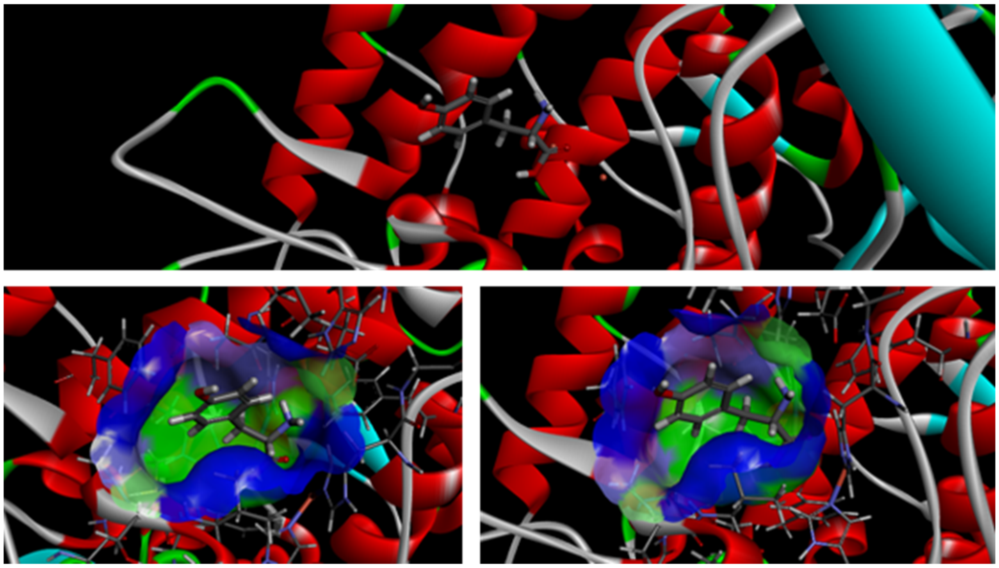

**Figure S14**. Pose No. 6 of tyrosine and tyrosinase docking. Red points: Cu; blue and green areas: solvent-accessible surfaces; red, gray, cyan, and green ribbons: tyrosinase.

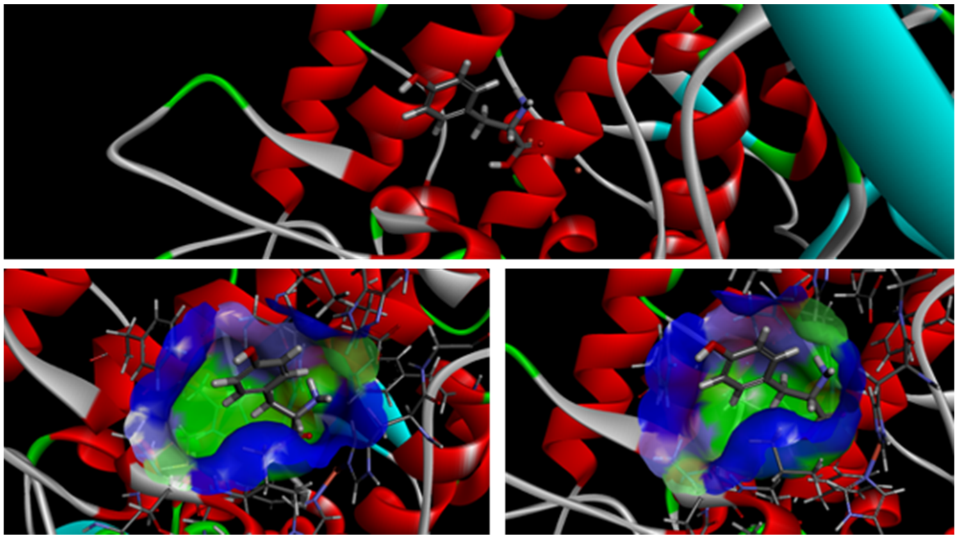

**Figure S15**. Pose No. 7 of tyrosine and tyrosinase docking. Red points: Cu; blue and green areas: solvent-accessible surfaces; red, gray, cyan, and green ribbons: tyrosinase.

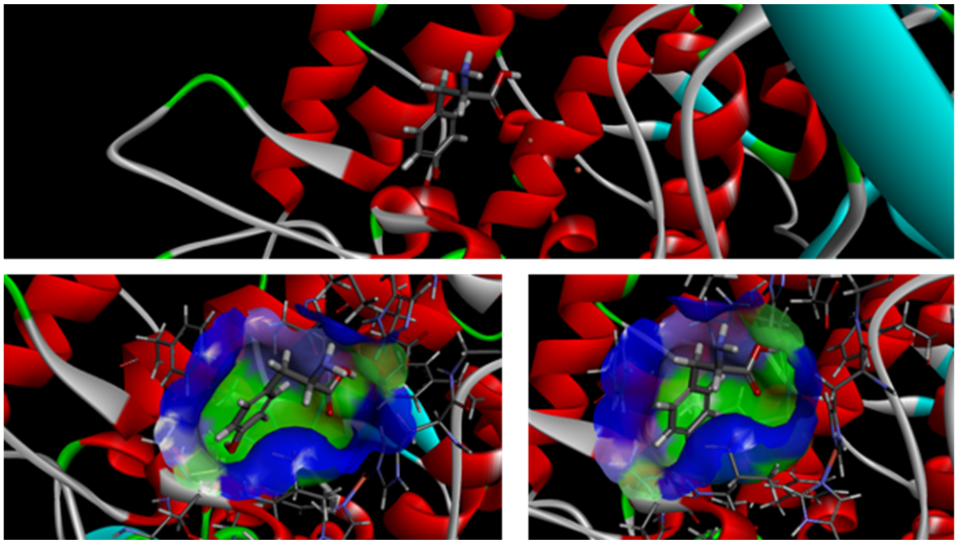

**Figure S16**. Pose No. 8 of tyrosine and tyrosinase docking. Red points: Cu; blue and green areas: solvent-accessible surfaces; red, gray, cyan, and green ribbons: tyrosinase.

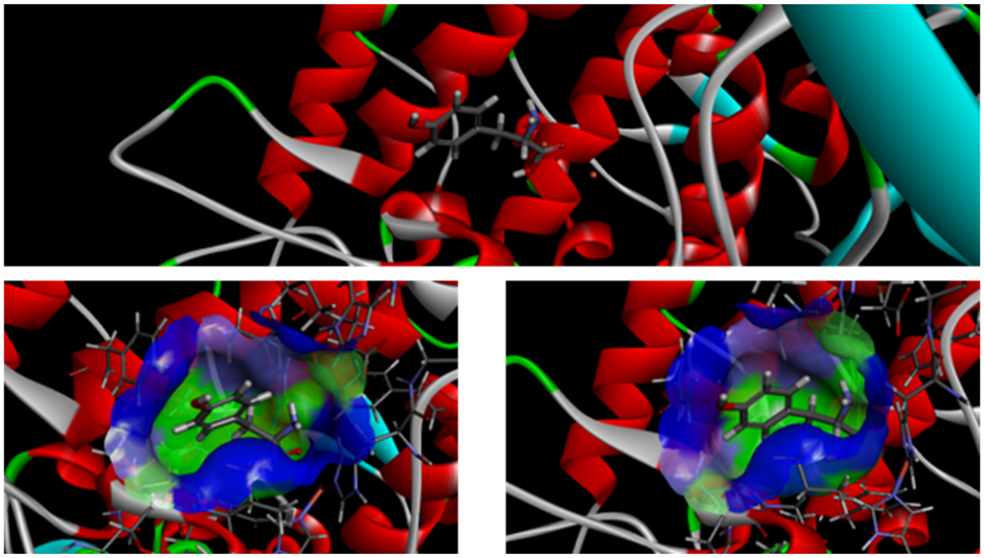

**Figure S17**. Pose No. 9 of tyrosine and tyrosinase docking. Red points: Cu; blue and green areas: solvent-accessible surfaces; red, gray, cyan, and green ribbons: tyrosinase.

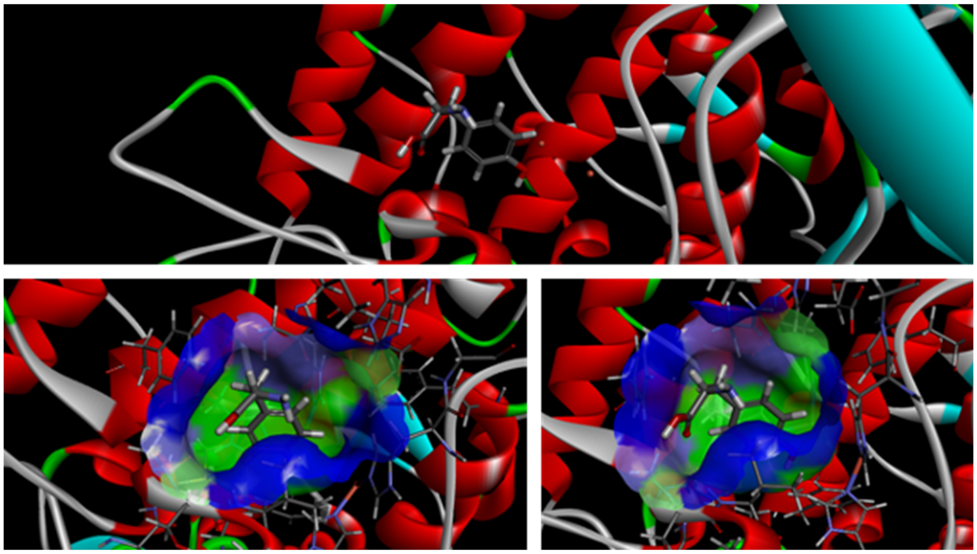

**Figure S18**. Pose No. 10 of tyrosine and tyrosinase docking. Red points: Cu; blue and green areas: solvent-accessible surfaces; red, gray, cyan, and green ribbons: tyrosinase.
